# Ocular leptospirosis: a review of current state of art of a neglected disease

**DOI:** 10.22336/rjo.2022.53

**Published:** 2022

**Authors:** César Elias Arrieta-Bechara, Angie Yesenia Carrascal-Maldonado

**Affiliations:** * Valladolid University, IOBA, Cadiz, Spain; ** Universidad del Bosque, Colombia, Bogota, Cadiz, Spain

**Keywords:** Leptospira, leptospirosis, leptospiral uveitis, ocular leptospirosis, Weil’s disease

## Abstract

**Introduction:** Leptospirosis is a neglected and re-emerging zoonotic disease that may cause ocular involvement, uveitis being the main complication of the systemic disease.

**Aim:** The purpose of this review was to raise awareness and update on uveitis caused by leptospirosis, which is a challenging pathology because it can mimic other types of uveitis.

**Materials and methods:** An article review, was conducted by searching PubMed (MEDLINE), Scielo, Cochrane Library databases using the following MeSH and DeCS terms: “leptospirosis”, “uveitis”, “ocular”, “eye” and “human”. The inclusion criteria were articles between 2000 and 2021 in English, Spanish, French or Portuguese.

**Results and Discussion:** A total of 49 articles were obtained with the mentioned inclusion criteria, and additionally 5 articles before the year 2000, which were considered due to their relevance and scarcity of articles on the pathology. Afterwards, a description of the disease was made.

**Conclusion:** This literature review was steered to raise awareness and to apprise physicians and ophthalmologists about a pathology that is becoming increasingly relevant, but underdiagnosed, even in developed countries.

**Abbreviations:** REU = recurrent equine uveitis, MAT = microagglutination

## Introduction

Leptospirosis is a zoonosis of worldwide distribution, more endemic in some regions of the world. It is caused by *Leptospira interrogans* and is a public health issue because it is a neglected [**1**] and re-emerging disease affecting humans [**2**,**3**].

In 1803, William Wittmen was the first to perform a clinical report of leptospirosis and later, in 1886, Weil described the jaundice component of the disease, and the entity was coined with his name. Later, in 1917, Noguchi first isolated the organism from a Norwegian rat. Leptospirosis uveitis was initially reported in an original paper by Weil [**4**].

The systemic disease has an extremely broad spectrum, ranging from an influenza syndrome to the presence of Weil’s disease including, fever, haemorrhage, acute liver and renal failure. Laboratory tests are required to confirm the diagnosis [**5**].

In addition to causing systemic infection, after a period of latency, leptospirosis may result in an immune disorder [**6**]. The ocular involvement in humans can range from non-specific congestion to scleral jaundice and uveitis, which is a complication of systemic disease [**7**]. 

Leptospirosis can affect animals varying from domestic to wild. In animals, however, leptospirosis-associated uveitis only occurs in horses, and is known as recurrent equine uveitis (REU) [**8**]. REU is one of the main causes of vision loss and is usually bilateral and has a major impact on financial costs, owners and animal health, often resulting in euthanasia [**9**]. REU has been used as a natural model for the study of autoimmune uveitis in humans, and has provided insight into endogenous factors of how the blood-ocular barrier is destroyed and initiates cellular and humoral processes that lead to vision loss [**8**].

## Aim

The purpose of this research was to highlight leptospirosis and its ocular involvement of neglected and re-emerging pathology that may cause ocular morbidity and severe sequels leading to decreased quality of life.

## Materials and methods

To carry out this review, an exhaustive bibliographic search was performed on MEDLINE (PubMed), Scielo, Cochrane Library databases using the following MeSH and DeCS terms: “leptospirosis”, “uveitis”, “ocular”, “eye” and “human”.

The inclusion criteria were: articles that mentioned “leptospirosis”, “uveitis”, “ocular”, “eye” and “human” and that addressed the development of the pathology, in Spanish, English, Portuguese or French, between 2000 and 2021.

The exclusion criteria were: articles that only mentioned the pathology but did not develop it or articles on non-humans, articles in languages other than the ones in the inclusion criteria, as well as opinion articles and articles outside the mentioned timeline.

## Results and discussion

Titles and abstracts of articles dealing with the pathology and meeting other inclusion criteria were manually reviewed, and duplicates were excluded.

Regarding PubMed database, 10,992 articles were found using the MeSH terms “leptospirosis”, while 47 articles were found by narrowing the search using “uveitis”, “ocular”, “eye” and “human”. 5 articles published before 2000 were included due to their importance and scarcity of articles in the period covered. Regarding Scielo, 872 articles were found, and when the search was refined using the words “uveitis”, “ocular”, “eye” and “human”, 2 articles were found. Similarly, in the Cochrane Library, 5 articles were found using the MeSH term “leptospirosis”, and no articles were found when using “uveitis”, “ocular”, “eye” and “human”.

**Fig. 1:**
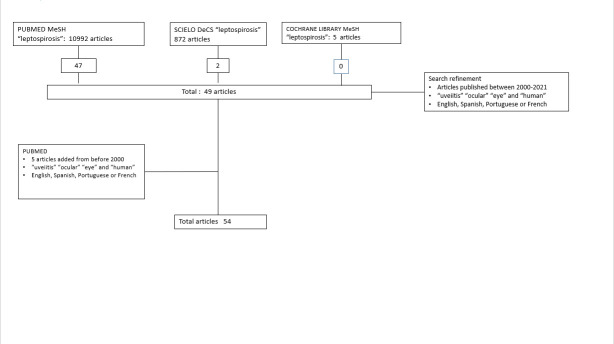
Data information

A total of 54 articles were found with the refinement of the search (**Fig. 1**).

*Etiology*

Leptospirosis is a zoonosis of worldwide distribution, caused by leptospira, a gram-negative spirochete of the genus *leptospira*, of which there are two species: *L. interrogans*, which causes the disease in humans, and *L. biflexia*, which is saprophytic. Twenty-three serogroups and more than 300 serovars have been identified. Leptospires are aerobic, motile, helicoidal, thin, axially filamentous bacteria with hooked ends, constantly rotating on their long axis [**4**,**10**].

In a retrospective study conducted in South India on 107 patients diagnosed with leptospiral uveitis by MAT (microagglutination), it was found that mixed infections were almost as frequent (48.5%) as infections with single organisms (50.4%) and Leptospira interrogans serovar australis had the highest overall incidence (35%) [**11**].

*Epidemiology and Transmission*

Regarding leptospirosis, it has been estimated that there are 1,000,000 cases per year worldwide and some authors, such as Wunder et al. [**10**] and Abela-Ridder et al. suggest that it accounts for 20-40% of cases of fever of unknown origin [**12**]. 

The disease is endemic in Asia, Oceania, and South America; however, cases are increasingly found in Europe and North America [**12**].

This is a re-emerging disease and therefore was not followed up in countries such as the United States, in which the incidence is unknown. However, it is known that there were 0.6 hospitalizations/ 1,000,000 people (95% CI 0.5-0.6) between 1998 and 2009 [**2**].

In China, Zhang et al. have reported an incidence of 0.7/ 100,000 people per year during the period 1991-2010. It was more frequent in men - 66.8%, compared to women - 33.2%, and especially in rural workers [**3**].

Conversely, Mancel et al. have described an incidence of leptospirosis as high as 180/ 100,000 inhabitants per year, in the islands of New Caledonia in the South Pacific [**5**]. Leptospirosis is one of the most frequently neglected diseases, as a consequence of under-reporting and lack of awareness of its existence [**13**]. In other Asian countries, such as Malaysia, it is considered to be endemic [**14**].

In European Union countries the incidence has been estimated at 1-4 cases/ million inhabitants depending on the country [**15**]. In the Netherlands, an incidence of 0.25 cases/ 100,000 inhabitants per year has been found in the period from 1925 to 2008 [**16**]. Meanwhile, in Germany, Jansen A et al. describe an incidence of 0.06 per 100,000 inhabitants during the period 1998-2003 [**17**]. The annual incidence in Spain was 0.86 cases per million inhabitants/ year during the period from 2009 to 2012 [**18**].

In Latin America, leptospirosis in humans in countries such as Argentina was estimated at 0.32/ 100,000 people in 2014 [**19**]. The main risk factors that have been associated with outbreaks of leptospirosis are flooding, the rainy season and hot weather [**20**]. In Colombia, the incidence has been estimated at between 1.1 and 5.4 cases per 100,000 inhabitants in the past decade [**21**]. In other countries such as Brazil, both urban and rural incidence is 1.9/ 100,000 people [**22**].

The vectors of leptospirosis are rodents, especially rats in urban environments. The bacterium usually remains in the kidneys of rodents for months and is then passed to humans via the urine of infected animals that contaminate water. It is most common in rubbish collectors, soldiers, milkmen, farm workers and travelers. The most common source of infection is urine from livestock, rodent, dogs or contaminated freshwater reservoirs. Cases have also occurred through infected blood. The disease is most prevalent in the tropics and subtropical areas [**23**]. Similarly, middle-aged men are more commonly affected. The main risk factor for acquiring the disease is exposure to water or soil contaminated with leptospires [**2**,**23**]. Other risk factors that have been associated with the pathology have been identified as areas with higher rainfall, forested areas especially in overpopulated regions, population in poverty, low educational level, vulnerable conditions and lack of sanitary structures [**21**, **24**].

The precise incidence of uveitis in systemic leptospirosis is unknown, but has been estimated to be between 3% and 92% depending on endemic or non-endemic areas [**6**].

*Pathogenesis*

The pathogenesis of leptospirosis is not well documented. Ninety percent of cases are asymptomatic. These are the initial cases or asymptomatic, or mild cases, in which a balance between inflammatory response and anti-inflammatory mechanisms will be observed, i.e., homeostasis will be maintained. In the other 10%, an imbalance between inflammatory response and anti-inflammatory mechanisms will be noted, leading to a large release of cytokines that will ultimately paralyze the immune response and lead to sepsis, multi-organ failure and death [**10**,**25**].

Its pathogenesis is not well known at the ocular level either, it is believed to be produced by the migration of antibodies to the anterior chamber, but there is a rapid decrease in these and the microorganism takes advantage of this to multiply [**26**].

It is also considered capable of releasing enzymes or bacterial toxins that injure tissues. Elevated levels of lipopolysaccharides have been found in aqueous humour in patients with leptospirosis uveitis, inducing congestion and increased vascular permeability. It has also been found that at a systemic level, high titers of TNF, Interleukin 6, 8, 10 can lead to immune-mediated vasculopathy due to activation and deposition of immunocomplexes. This leads to vascular endothelial damage and tissue injury [**25**,**27**,**28**]. 

Rapid progression to cataract has also been found in patients with uveitis due to leptospirosis and is believed to be due to leptospira that are cross-reactive against proteins that keep lens transparency [**29**]. Leptospiral lipoproteins coded by LruA and LruB in patients with leptospiral uveitis have been discovered in humans and LruA and LruB type IgG and IgA antibodies have also been found, which could have a cross-reaction against ocular components such as the retina, lens and ciliary body, although these antibodies can be seen in other types of uveitis such as Bechet’s [**29**,**30**]. 

*Systemic clinic*

The incubation period for leptospirosis is 3 to 30 days. The onset of symptoms is sudden-onset, biphasic fever. There is a septicaemia phase and an immune phase. The first period is a fever or septicaemia phase, followed by a fever-free period. This is followed by a second period of fever with or without jaundice or immune phase. In addition, other symptoms may be present: headache, retro-ocular pain, chills, chronic generalized fatigue and arthralgias [**31**].

It may also cause encephalitis, meningoencephalitis, transverse myelitis and cranial nerve palsies [**32**]. At the cardiac level, arrhythmias have been described, with P-Q delay, atrial fibrillation being quite common, as well as myocarditis and pericarditis [**23**]. Bal A describes gastrointestinal involvement, characterized by diarrhea, jaundice, vomiting, pancreatitis, and cholecystitis [**32**]. The main organ affected is the kidney, as the Leptospira has a tropism for the kidney, leading to acute renal failure. Other affections that could be seen are acute liver failure, pneumonitis, haemolytic anaemia, haemorrhagic diathesis, adrenal insufficiency, multisystem failure and death [**31**,**33**].

It could also have an involvement during pregnancy, from intrauterine growth retardation, foetal or placental ischaemia to much more severe consequences that can lead to foetal death and miscarriage, or congenital leptospirosis [**23**].

There are two terms that must be differentiated. The first one, is the most severe form of icteric leptospirosis that is called Weil’s syndrome. The second one, Weil’s disease, is an anicteric leptospirosis, renal failure and hepatic haemorrhage, multi-organ failure, vascular collapse and severe alterations in consciousness [**4**]. Systemic leptospirosis has a mortality of more than 10% in severe cases [**10**]. Cagliero J et al. have estimated that more than 60,000 deaths occur annually from leptospirosis [**25**].

*Ocular involvement*

The incidence of ocular signs varies from 2% to 90% during the acute phase. Ocular signs tend to occur in the immune phase [**4**,**18**,**34**].

One of the early manifestations is chemosis, which may be as high as 85.7% and usually accompanies systemic manifestations. Sub-conjunctival haemorrhage may also occur in 19.5% of cases. Other clinical features of ophthalmological involvement are: cataract in 14% with rapid progression and spontaneous reabsorption, and glaucoma [**4**,**6**,**26**,**32**]. Rathinam et al. analyzed 394 eyes of 276 patients with seropositive leptospiral uveitis and found that 13.7% developed cataracts between 1-6 months after presentation and, of these, spontaneous absorption was observed in 18.5% with a median of 5 months after cataract onset [**35**]. Another article that included 6 young patients with leptospirosis, underlined an early onset and rapid progression to cataract formation within a period of three months [**36**].

The presence of icteric sclera and pericorneal congestion is considered pathognomonic of severe systemic leptospirosis. Interstitial keratitis may also be present in up to 18% [**6**]. Rathinam et al. also reported a case of corneal melting secondary to ocular infection due to leptospirosis [**37**].

Among the late findings, the most important is the uveitis. This is the most frequent ocular complication of the systemic disease. It can be unilateral or bilateral [**38**,**39**] and it occurs in 40% of patients with systemic disease [**30**]. It usually occurs in two weeks after the infection up to 1 year later, with an average duration of 6 months. Uveitis can be anterior uveitis or panuveitis. The most common is anterior uveitis. Its course is usually insidious and mild [**40**,**41**]. It may present with hypopyon in 2.7% to 12% of cases, and may be non-granulomatous or much more rarely granulomatous [**26**,**32**,**42**]. 

The other form, as it usually manifests itself, is as acute and severe panuveitis. In a study by Rathinam et al., which included 73 patients and 111 eyes with leptospirosis uveitis, panuveitis was found in 95.5% of the eyes. The most frequent findings were non-granulomatous reactions in 91.9%, keratic precipitates - 85.6%, vitritis in 76.6% of eyes, periphlebitis - 51.4%, posterior synechiae - 23.4%, hypopyon - 12.6%, choroiditis - 4.5%, papillitis - 3.6%, macular oedema - 2.7%, epiretinal membrane - 1.8%, pars planitis - 1.8% and arteritis - 0.9% [**38**]. 

In a newer article from 2005, Rathinam SR described optic nerve hyperaemia in leptospirosis uveitis as ranging from 3% to 64%. He also reported neuroretinitis, optic neuritis and retrobulbar neuritis [**6**].

Martins et al. in Brazil described the ocular manifestations of leptospiral uveitis in 21 patients with conjunctivitis - 85.7%, increased retinal venous caliber - 57.1%, optic disc hyperaemia - 57.1%, and sub-conjunctival haemorrhage - 19.0%. Also, optic disc oedema, retinal vasculitis, retinal haemorrhage, hard exudates and papillitis were reported in 4.8% each [**42**]. Moreover, Costa et al. in Brazil described a patient with sickle cell anaemia who presented with massive bilateral vitreous haemorrhage with retinal detachment and phthisis bulbis in the context of leptospiral infection [**43**].

Similarly, rare cases of bilateral sixth cranial nerve palsies and acute bilateral keratouveitis have been reported [**44**,**45**]. 

In a much more recent study undergone in 2016, Sivakumar R et al. reported that non-granulomatous panuveitis, hypopyon, and vitreous infiltration in the absence of retinochoroiditis are predictive parameters for leptospirosis uveitis with 86% sensitivity and 90.7% specificity [**7**].

*Diagnosis*

Diagnosis for systemic diseases can be made by laboratory tests that identify the spirochete directly, or indirectly by tests that show the presence of infection. It can be performed on blood, urine or cerebrospinal fluid. There are four groups of main tests. Being the first one, direct tests, which isolate the bacteria most frequently during the septicaemic phase of the disease, include darkfield microscopy, silver staining, immunohistochemical staining, and immunoperoxidase staining. A second group of tests is animal inoculation [**6**].

The third group includes serological tests such as the macroscopic agglutination test, MAT test, haemagglutination test and the ELISA test. The latter test is rapid and commercially available and can have good sensitivity and specificity. Immunoglobulin G ELISA has been used. In addition, Immunoglobulin M ELISA, which can be detected 4 days after symptoms and can persist for up to 5 months, has a sensitivity of 88% and specificity of 96%. MAT, in which titers or concentrations greater than 1:400 are considered positive, and in chronic disease, values greater than 1:100 signify an infection, is also used [**6**]. According to the World Health Organization, MAT is the gold standard for the diagnosis of leptospirosis. It has the disadvantage that it requires a specialized laboratory and trained personnel [**46**,**47**].

The last group is represented by molecular biology techniques like PCR, which is recommended when there is uncertainty with a MAT test or an ELISA test [**5**,**46**,**47**]. Haake et al. described the finding of 80% Leptospira DNA in aqueous humour by DNA enzymatic PCR, pulsed-field electrophoresis and restriction fragment length polymorphism techniques [**31**]. 

However, there in not a specific method for ocular diagnosis [**6**]. MAT detects uveitis due to leptospirosis in 50% of cases [**30**]. MAT and IgM ELISA together are positive for leptospirosis uveitis in 77% cases [**29**]. The use of lipopolysaccharides for the diagnosis of leptospiral uveitis is also being studied [**48**]. An article reported a sensitivity of 48% and a specificity of 90% with lipopolysaccharides [**29**]. Kits such as Lepto IgM MICROLISA and Leptocheck (Leptospira IgM Antibodies) have been tested for the diagnosis of leptospiral uveitis, but the sensitivity and specificity in the first was 60% and 55%, and for the second 80% and 59% respectively, having these results limitations for their use [**34**]. Due to leptospirosis, a protein produced by the bacterium, called HbpA, has been detected in uveitis. Anti-HbpA Ig are produced against this protein, which have been detected in 92% of patients with uveitis due to leptospirosis, being more sensitive than MAT and without cross-reaction like other types of uveitis [**49**].

**Table 1:** Characteristics of ocular involvement for leptospirosis

Leptospirosis ocular involvement	Clinical manifestations
Anterior uveitis	• Insidious and mild hypopyon [**26**]
	• Non-granulomatous or much more rarely granulomatous [**25**,**29**,**32**]
Panuveitis	• Non-granulomatous keratic precipitates, vitritis, periphlebitis, posterior synechiae, hypopyon choroiditis, papillitis, macular oedema, epiretinal membrane, pars planitis [**38**]
	• Arteritis 0.9% [**31**]
	• Increased retinal venous caliber [**38**]
Other anterior segment findings	• Conjunctival hyperaemia [**49**]
	• Corneal melting [**37**]
	• Chemosis, conjunctival haemorrhage, cataract with rapid progression and spontaneous reabsorption, icteric sclera and pericorneal congestion (pathognomonic), interstitial keratitis [**4**,**6**,**26**,**32**]
	• Acute bilateral keratouveitis [**45**]
	• Bilateral massive vitreous haemorrhage with retinal detachments and phthisis bulbis [**43**]
Optic nerve	• Glaucoma [**4**,**6**,**26**,**32**]
	• Neuroretinitis, optic neuritis and retrobulbar neuritis [**6**]
	• Papillitis [**42**]
Others	• Sixth cranial nerve palsies [**44**]

*Differential diagnosis*

Systemic differential diagnoses should be suggested when the patient is living in an endemic area and what should also be considered are influenza, rhizocystis disease, malaria, or typhoid [**50**].

Leptospirosis uveitis is a pathology that is underdiagnosed and can simulate another type of uveitis. Among the uveitis with which a differential diagnosis must be made are HLA B27 uveitis, Adamantides-Behcet disease, both of which can present with hypopyon. Also, the other types of uveitis are sarcoidosis and endogenous endophthalmitis [**6**], tuberculosis [**44**] or syphilis, brucellosis, or Eales disease [**4**].

*Treatment*

The treatment of severe systemic leptospirosis is performed with penicillin G, 1.5 units every 6 hours for one week [**51**]. Cephalosporins such as ceftriaxone and cefotaxime have also been used for severe forms [**52**]. If the infection is mild to moderate, doxycycline can be used every 12 hours for one week [**51**].

Regarding the Jarisch-Herxheimer reaction, which occurs in infections caused by spirochetes, there is controversy, but it is thought to be less prevalent in leptospirosis than in other spirochetes [**4**].

Anterior uveitis is usually self-limiting and resolves with the help of corticosteroids and mydriatics [**4**]. However, there are no clinical trials supporting antibiotic treatment in leptospirosis uveitis. In cases of leptospirosis panuveitis, topical, periocular or systemic corticosteroids should be added in addition to antibiotic treatment [**51**]. 

Work is ongoing on developing a possible vaccine. LipL45 (lipopolysaccharide DNA) vaccine was used in mice and it induced a significant Th1 type immune response [**53**].

*Prognosis*

The mortality of systemic leptospirosis varies from 1% to 20% depending on the severity of the disease, the latter figure being a consequence of multi-organ failure [**54**].

In leptospirosis uveitis, the prognosis is usually good despite the severe visual compromise that patients may have; patients recover their vision once the uveitis resolves [**4**].

## Conclusion

To sum up, it can be stated that leptospirosis and leptospiral uveitis are globally distributed, neglected and re-emerging pathologies and the aim of the present article was to make them visible and to make doctors and especially ophthalmologists aware of these pathologies.

**Conflict of Interest statement**

The authors state no conflict of interest.

**Acknowledgements**

None.

**Sources of Funding**

None.

**Disclosures**

None.
